# Chitin Oligosaccharide Modulates Gut Microbiota and Attenuates High-Fat-Diet-Induced Metabolic Syndrome in Mice

**DOI:** 10.3390/md16020066

**Published:** 2018-02-19

**Authors:** Junping Zheng, Gong Cheng, Qiongyu Li, Siming Jiao, Cui Feng, Xiaoming Zhao, Heng Yin, Yuguang Du, Hongtao Liu

**Affiliations:** 1 Liaoning Provincial Key Laboratory of Carbohydrates, Dalian Institute of Chemical Physics, Chinese Academy of Sciences, Dalian 116023, China; junpingzheng2013@163.com (J.Z.); zhaoxm@dicp.ac.cn (X.Z.); yinheng@dicp.ac.cn (H.Y.); 2 University of Chinese Academy of Sciences, Beijing 100049, China; 3 State Key Laboratory of Biochemical Engineering and Key Laboratory of Biopharmaceutical Production & Formulation Engineering, PLA, Institute of Process Engineering, Chinese Academy of Sciences, Beijing 100190, China; gcheng@ipe.ac.cn (G.C.); qiongyulee@hotmail.com (Q.L.); smjiao@ipe.ac.cn (S.J.); cfeng@ipe.ac.cn (C.F.); 4 Zhengzhou Institute of Emerging Industrial Technology, Zhengzhou 450000, China

**Keywords:** chitin oligosaccharide, gut microbiota, high-fat diet, insulin resistance, metabolic syndrome

## Abstract

Gut microbiota has been proved to be an indispensable link between nutrient excess and metabolic syndrome, and chitin oligosaccharide (NACOS) has displayed therapeutic effects on multiple diseases such as cancer and gastritis. In this study, we aim to confirm whether NACOS can ameliorate high-fat diet (HFD)-induced metabolic syndrome by rebuilding the structure of the gut microbiota community. Male C57BL/6J mice fed with HFD were treated with NACOS (1 mg/mL) in drinking water for five months. The results indicate that NACOS improved glucose metabolic disorder in HFD-fed mice and suppressed mRNA expression of the protein regulators related to lipogenesis, gluconeogenesis, adipocyte differentiation, and inflammation in adipose tissues. Additionally, NACOS inhibited the destruction of the gut barrier in HFD-treated mice. Furthermore, 16S ribosome RNA sequencing of fecal samples demonstrates that NACOS promoted the growth of beneficial intestinal bacteria remarkably and decreased the abundance of inflammogenic taxa. In summary, NACOS partly rebuilt the microbial community and improved the metabolic syndrome of HFD-fed mice. These data confirm the preventive effects of NACOS on nutrient excess-related metabolic diseases.

## 1. Introduction

Metabolic syndrome, characterized by at least three of five medical conditions (obesity, hypertension, hyperglycemia, insulin resistance, and dyslipidemia), quintuples the risk of type 2 diabetes mellitus [1]. Increasing data indicate that gut microbiota is necessary to maintain the metabolic homeostasis of a host [2]. For instance, germ-free mice are resistant to high-fat diet (HFD)-induced obesity [3], but are prone to gain weight after fecal transplantation from obese mice [4]. It was also reported that the population of pro-inflammatory bacteria was elevated, and the abundance of beneficial bacteria was decreased, in the gut of obese or diabetic mice [5,6,7]. Furthermore, it was proved that several beneficial bacteria, such as *Bifidobacterium*, *Lactobacillus*, and *Akkermansia*, independently ameliorated hyperglycemia, glucose intolerance, and insulin resistance in HFD-fed mice [8,9,10,11].

The gut barrier, which is mainly composed of mucus layers and tight junctions, is an indispensable structure that maintains the balance between hosts and their microbiome [12]. The mucus layer above epithelial cells is formed by glycosylated mucin [13]. During gut dysbiosis, the degradation of mucin is observed and is accompanied by a decreased abundance of *Akkermansia muciniphila*, which is an intestinal mucin-degrading bacterium and is involved in the maintenance of the mucus layer [9,12]. The tight junction is under the mucus layer and its integrity is mainly regulated by zonula occludens-1 (ZO-1) and occludin. Evidence shows that the levels of ZO-1 and occludin in jejunum epithelia are reduced in ob/ob mice [14]. Noticeably, the corruption of each layer of the gut barrier can cause serious outcomes. Lipopolysaccharides (LPS), produced by intestinal bacteria, can easily penetrate into the circulatory system through a leaky barrier. The leaked LPS can trigger adipocyte inflammation and eventually lead to insulin resistance, or even diabetes [15]. Adipocyte inflammation is characterized by the transcriptional upregulation of pro-inflammatory cytokines, including COX-2, IL-6, MCP-1, and TNF-α [16].

One of the strategies for improving metabolic syndrome is to maintain the balance of the gut ecosystem [17]. To date, there have been a series of prebiotics developed to regulate the intestinal microbial community. Among them, galacto- and xylo-oligosaccharides have been found to boost the abundance of beneficial bacteria (*Bifidobacterium*, *Lactobacillus*, and *Akkermansia*) [18,19], and to improve the metabolic profile in HFD mice [20]. In comparison with expensive, first-line medicines for diabetic treatment, oligosaccharides are usually produced at a low cost and show non- or less toxicity in vivo. Thus, oligosaccharide prebiotics should play a critical role in preventing against metabolic diseases. 

Chitin, a cellulose-like polysaccharide linked by β 1-4 *N*-acetylglucosamine, is present in the exoskeleton of crab and shrimp [21,22]. Via chitinase digestion, chitin oligosaccharide (*N*-acetyl-chitooligosaccharide, NACOS) can be produced and further deacetylated into chitosan oligosaccharide (COS) [23]. COS with a low molecular weight (<1000 Da) was reported to markedly inhibit glucose uptake by the intestine tract by suppressing the activities of pancreatic α-amylase and small intestinal α-glucosidase [24,25]. Additionally, COS increases insulin secretion by promoting the antioxidant capacity of the pancreas [26,27], and exerts anti-diabetic effects in db/db mice and streptozotocin-injected rats [28]. Especially, COS displayed low subacute toxicity and no adverse effect in rats at large dosage [29]. As compared to the investigations on COS, less work has been done regarding NACOS. Thus far, limited studies suggest that NACOS can be developed as a potential functional food, or biological medicine, for the treatment of multiple diseases, such as cancer, gastritis, and Alzheimer’s disease [30,31,32]. In addition, chitin–glucan fiber can ameliorate metabolic disorder in HFD-fed mice [33]. Furthermore, NACOS increased the ratio of *Lactobacillus*/*Enterococcus* in human fecal culture [34]. However, it remains to be seen whether NACOS can improve metabolic syndrome induced by nutritional excess.

Based on the above, we hypothesize that NACOS can alleviate metabolic disorder by modulating gut microbiota in HFD-fed mice. In this study, we assessed the suppressive effect of NACOS on HFD-induced disturbance in lipogenesis, gluconeogenesis, and inflammation in mice. Additionally, we explored the reshaping of the structure of unbalanced gut microbiota after NACOS treatment.

## 2. Results

### 2.1. Effect of NACOS on Food Intake, Drinking Water, and Body Composition of HFD-Fed Mice

**Figure 1:**
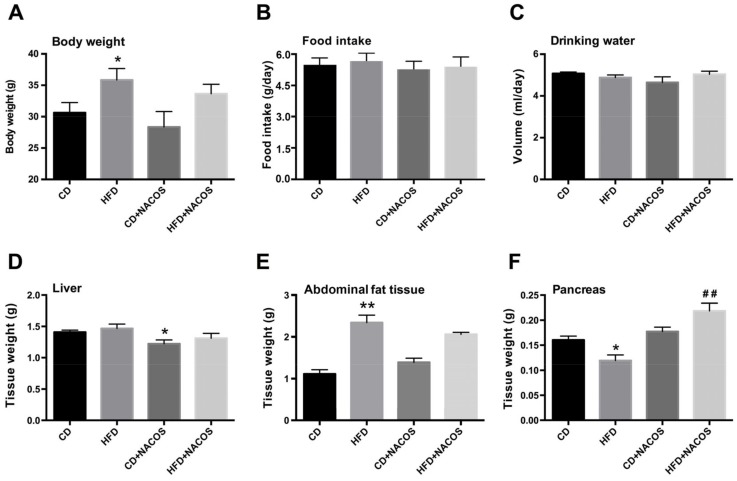
Effects of chitin oligosaccharide (NACOS) on body composition of high-fat diet (HFD)-fed mice. Mice were fed with normal chow diet (CD), HFD, CD + NACOS (1 mg/mL, in drinking water, about 200 mg/kg/day) or HFD + NACOS for five months. During the treatment period, body weight (**A**), diet consumption (**B**) and water drinking (**C**) were monitored for each group. After that, mice were euthanized for tissue collection, and the weights of liver (**D**), abdominal fat tissue (**E**), and pancreas (**F**) were detected. Data are represented as means ± SD (*n* = 5). * *p* < 0.05, ** *p* < 0.01 compared to the CD group; ^##^ *p* < 0.01 compared to the HFD group.

To evaluate the effects of NACOS on metabolic syndrome, C57BL/6J mice were fed with HFD and were spontaneously treated with either a vehicle (sterile tap water) or NACOS. Referring to previous studies of NACOS and COS in mice [35,36], the concentration of 1 mg/mL NACOS (around 200 mg/kg/day, in drinking water) was used in this study. As compared with the CD group, the HFD group displayed an evidently higher body weight, which was prone to decrease after NACOS treatment (Figure 1A). During the treatment period, no significant differences in food intake and water drinking were observed among all groups (Figure 1B,C). On the other hand, NACOS feeding had no effect on the weight of liver or abdominal fat tissue in HFD-fed mice (Figure 1D,E). In addition, HFD feeding led to a dramatic decrease in pancreas mass (*p* < 0.05, vs. the CD group), which was greatly reversed using NACOS treatment (*p* < 0.01, vs. the HFD group) (Figure 1F).

### 2.2. NACOS Ameliorated Glucose Intolerance and Increased Insulin Secretion of HFD-Treated Mice

**Figure 2:**
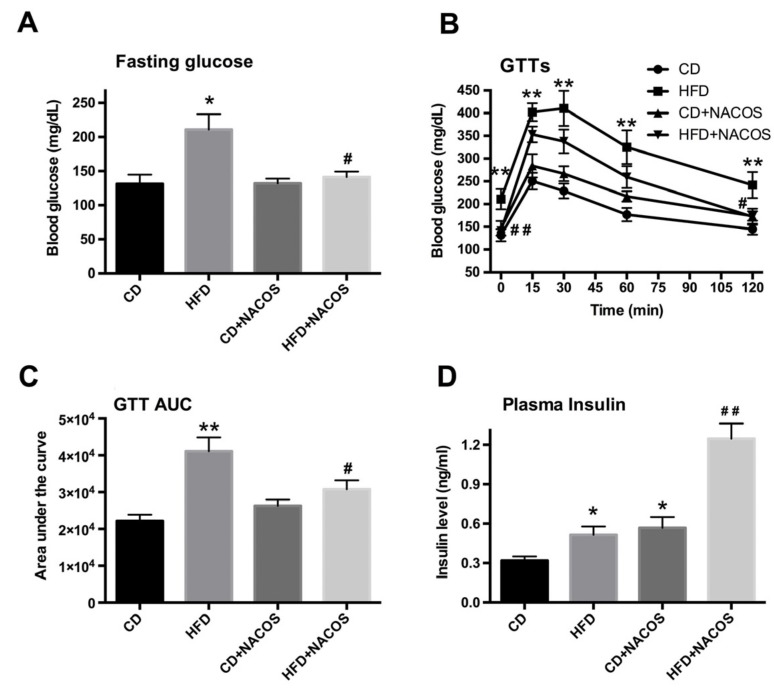
Effects of NACOS on glucose intolerance and insulin secretion of HFD-treated mice. Mice were fed with CD, HFD, CD + NACOS (1 mg/mL, in drinking water, about 200 mg/kg/day), or HFD + NACOS for five months. During the treatment period, fasting glucose (**A**) was measured. After that, a glucose tolerance test (**B**) was conducted and the areas under the glucose response curve (**C**) were calculated at the indicated time points (0–120 min) after intraperitoneal injection of glucose (2 g/kg·body weight). Finally, all mice were euthanized and plasma was collected for random insulin detection (**D**) using ELISA. Data are represented as means ± SD (*n* = 5). * *p* < 0.05, ** *p* < 0.01 compared to the CD group; ^#^ *p* < 0.05, ^##^ *p* < 0.01 compared to the HFD group.

During the treatment period, fasting glucose was monitored frequently until the hyperglycemic symptom was obvious in the HFD-fed group (*p* < 0.05, vs. the CD group) (Figure 2A). Compared with the HFD group, mice fed with HFD plus NACOS had a lower fasting glucose, which was nearly the equivalent of that in the CD group (Figure 2A). Further, a glucose tolerance test was carried out after fasting for 12 h. Blood glucose was detected at indicated time points (0 min, 15 min, 30 min, 60 min, and 120 min) after intraperitoneal injection of glucose (2 g/kg body weight). As indicated in Figure 2B, the mice in the HFD group showed poorer behavior in terms of glucose tolerance at individual time points relative to that in the CD group, which was significantly improved by NACOS feeding. A similar result was found with respect to the area under the blood glucose response curve (Figure 2C). Next, the plasma insulin of each mouse was measured and it turned out that NACOS feeding strikingly promoted insulin secretion; notably higher than that of the HFD group (*p* < 0.01, vs. the HFD group).

### 2.3. NACOS Alleviated Dyslipidemia in HFD-Fed Mice

**Figure 3:**
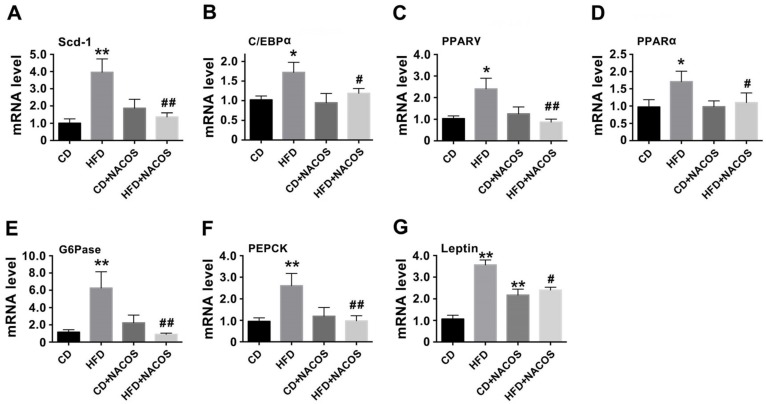
Effects of NACOS on lipid/glucose metabolism-related markers at the transcription level in the abdominal adipose tissues of HFD-treated mice. Mice were fed with CD, HFD, CD + NACOS (1 mg/mL, in drinking water, about 200 mg/kg/day), or HFD + NACOS for five months. After treatment, mice were euthanized and the abdominal adipose tissues were collected for mRNA detection of lipid/glucose metabolism-related markers by RT-PCR analysis, including Scd-1 (**A**), C/EBPα (**B**), PPARγ (**C**), PPARα (**D**), G6Pase (**E**), PEPCK (**F**), and leptin (**G**). Data are represented as means ± SD (*n* = 5). * *p* < 0.05, ** *p* < 0.01 compared to the CD group; ^#^ *p* < 0.05, ^##^ *p* < 0.01 compared to the HFD group.

To determine how NACOS regulated the lipid or glucose metabolism of HFD-treated mice, the lipids in blood and liver tissues were determined (Figure S4). The results suggest that NACOS treatment significantly decreased HFD-induced hyper-triglyceride (Figure S4A,B) and reversed the decrease of high-density lipoprotein-cholesterol (HDL) in HFD-fed mice (Figure S4C). Further, abdominal fat tissues were collected for the analysis of lipogenesis protein regulators (Scd-1, stearoyl-CoA desaturase-1), adipocyte differentiation (C/EBPα, CCAAT/enhancer-binding protein alpha; PPARα, peroxisome proliferator-activated receptor alpha; PPARγ, peroxisome proliferator-activated receptor gamma), and gluconeogenesis (G6Pase, glucose-6-phosphatase; PEPCK, phosphoenolpyruvate carboxykinase). We found that NACOS treatment significantly inhibited up-regulation of the mentioned genes in HFD-treated mice at the mRNA level (*p* < 0.05 or 0.01, vs. the HFD group) (Figure 3A–F). Furthermore, NACOS suppressed HFD-induced over-expression of leptin mRNA in adipose tissue (*p* < 0.05, vs. the HFD group) (Figure 3G). Additionally, NACOS profoundly inhibited lipid accumulation in the liver (Figure S5) and reversed the hypertrophy of adipocytes in HFD-fed mice (Figure S6).

### 2.4. NACOS Attenuated Inflammation in HFD-Fed Mice

**Figure 4:**
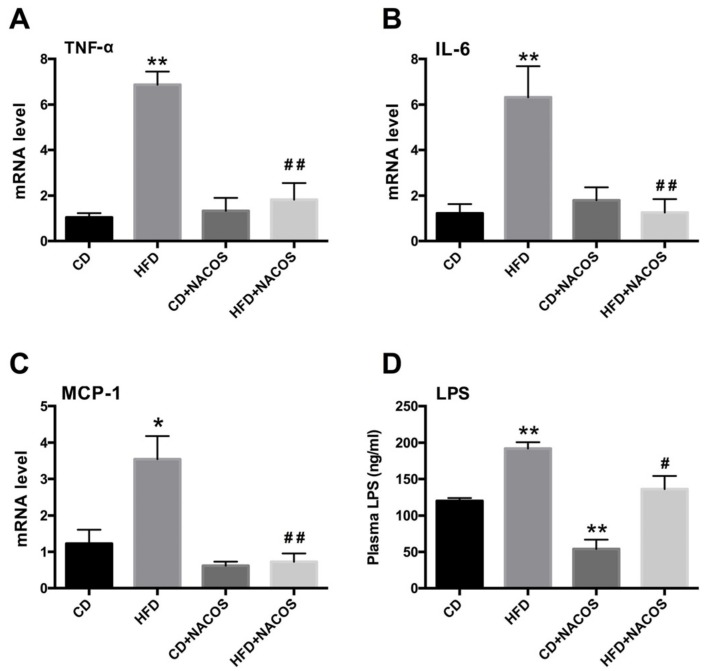
Inhibition of inflammatory responses in abdominal adipose tissue and decrease of plasma LPS in HFD-fed mice by NACOS treatment. Mice were fed with CD, HFD, CD + NACOS (1 mg/mL, in drinking water, about 200 mg/kg/day), or HFD + NACOS for five months. After the treatment, mice were euthanized. The abdominal adipose tissues were collected for mRNA detection of pro-inflammatory cytokines by RT-PCR analysis, including TNF-α (**A**), IL-6 (**B**), and MCP-1 (**C**). The blood was collected for LPS detection (**D**) by ELISA. Data are represented as means ± SD (*n* = 5). * *p* < 0.05, ** *p* < 0.01 compared to the CD group; ^#^ *p* < 0.05, ^##^ *p* < 0.01 compared to the HFD group.

Inflammation usually leads to dyslipidemia of adipocyte tissue [37]. In the present study, the mRNA expression of pro-inflammatory cytokines in adipose tissues was analyzed. As shown in Figure 4, significantly increased levels of tumor necrosis factor alpha (TNF-α), interleukin 6 (IL-6), and monocyte chemoattractant protein 1 (MCP-1) were observed in the HFD group, which were strongly reduced by NACOS treatment (*p* < 0.05 or 0.01, vs. the HFD group) (Figure 4A–C). We also monitored LPS levels in the circulatory system, and the result suggests that NACOS significantly decreased the blood concentration of LPS in HFD-fed mice (Figure 4D). 

### 2.5. NACOS Protected Intestinal Integrity in HFD-Fed Mice

**Figure 5:**
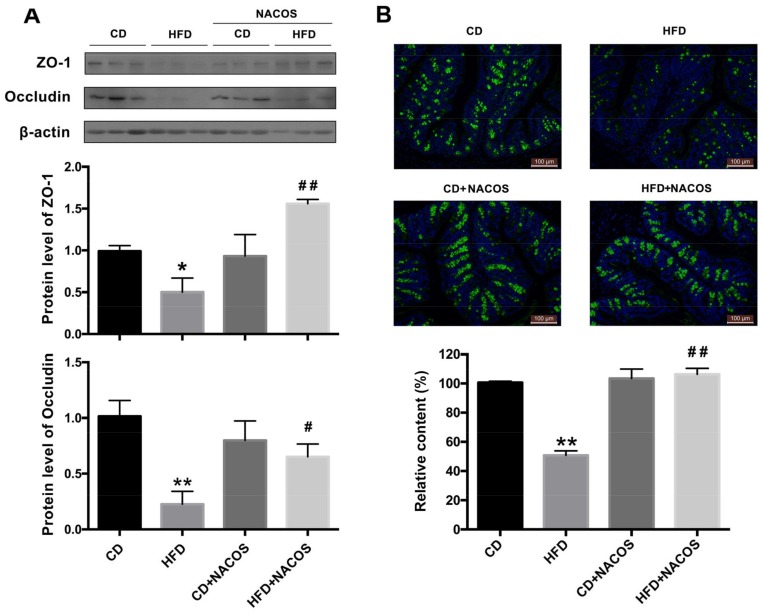
Improvement of gut barrier damage of HFD-fed mice by NACOS administration. Mice were fed with CD, HFD, CD + NACOS (1 mg/mL, in drinking water, about 200 mg/kg/day), or HFD + NACOS for five months. After treatment, mice were euthanized and the intestinal segments (jejunum and colon) were collected for further analysis. (**A**) Effects of NACOS on the protein expression of ZO-1 and occludin in intestinal tissues of HFD-fed mice by Western blot analysis. (**B**) Effect of NACOS on the damage to intestinal mucus layer in HFD-fed mice by WGA-FITC staining. The contents of mucin glycoproteins were reflected by the fluorescence intensity of each slide. Green fluorescence, mucin glycoprotein staining; blue fluorescence, nucleus staining. Data are represented as means ± SD (*n* = 5). * *p* < 0.05, ** *p* < 0.01 compared to the CD group; ^#^ *p* < 0.05, ^##^ *p* < 0.01 compared to the HFD group.

Evidence shows that low-grade inflammation may be attributed to the impairment of gut integrity [38]. To study the role of NACOS in modulating the intestinal tight junction of HFD-fed mice, the proximal colon was collected for the detection of major tight junction proteins, including ZO-1 and occludin, via Western blot analysis. Results show that the protein levels of both ZO-1 and occludin were considerably decreased in the HFD group, and were partly normalized by NACOS feeding (*p* < 0.05, vs. the HFD group) (Figure 5A). Furthermore, the mucus layer was stained with wheat germ agglutinin labeled with FITC (WGA-FITC) to visualize the impairment of the intestinal barrier. Green signals represented mucin glycoproteins and blue signals showed nucleus location using DAPI staining. Results indicated that NACOS significantly inhibited the decrease in green fluorescence intensity of HFD-fed mice, suggesting that NACOS treatment prevented damage to the intestinal mucus layer (Figure 5B). 

### 2.6. NACOS Protected Gut Microbiota from Destruction in HFD-Fed Mice

**Figure 6:**
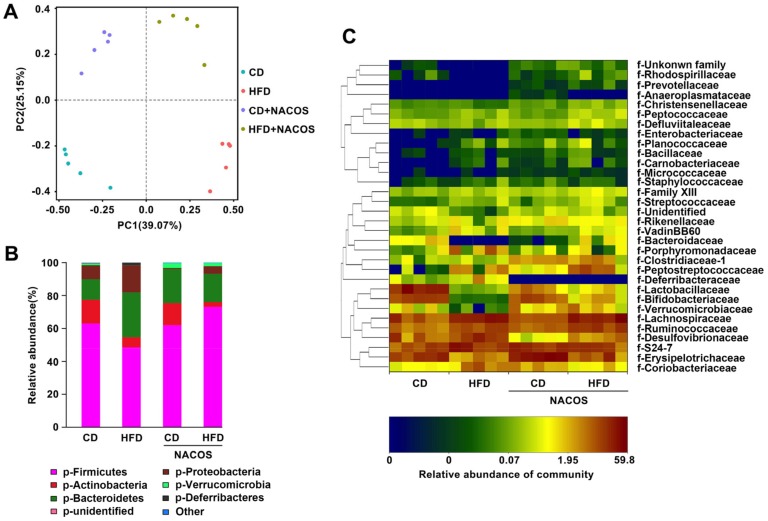
Inhibitory effects of NACOS on the imbalance of gut microbiota of HFD-fed mice. Mice were fed with CD, HFD, CD + NACOS (1 mg/mL, in drinking water, about 200 mg/kg/day), or HFD + NACOS for five months. After the treatment, mice were euthanized and the fecal contents were collected for 16S rRNA sequence analysis. (**A**) Bacterial community assay using the principal component analysis; (**B**) bar plot of microbial community at phylum level; (**C**) relative abundance of each classified family using heat map analysis.

Increased intestinal permeability is generally caused by gut dysbiosis. To unveil the protective effect of NACOS on maintenance of gut microbiota composition in HFD-fed mice, 16S rRNA gene sequencing of fecal samples was carried out. Remarkably, principal component analysis based on operational taxonomic unit (OTU) abundance indicated that the microbial composition was distinctly separated among all experimental groups. Further, the relative abundance of bacteria at the phylum level was obviously different (Figure 6B). The ratio of Fimicutes/Bacteroidetes was statistically decreased in the HFD group, which was almost completely restored in the HFD+NACOS group (Figure 6B). On the other hand, NACOS feeding profoundly suppressed the growth of Proteobacteria induced by HFD (Figure 6B). In addition, NACOS feeding slightly increased the abundance of Verrucomicrobia in comparison with the HFD group (Figure 6B). 

Next, to determine which bacterial families shaped the distinct microbiota structures between four groups, the relative abundances of bacteria families were tabulated on a heat map (Figure 6C). It was found that 11 families displayed significantly different abundances between the HFD group and the HFD + NACOS group, indicating that the protective effect of NACOS on mouse metabolic syndrome may be mediated by a subset of bacterial taxa. The relative abundances of Rhodospirillaceae, Christensenellaceae, Bacteroidaceae, Lactobacillaceae, Bifidobacteriaceae, Verrucomicrobiaceae, and Erysipelotrichaceae were decreased by HFD treatment, while NACOS rescued these HFD-induced changes to a large extent. Contrary to this, HFD treatment increased the abundances of Porphyromonadaceae, Deferribacteraceae, Desulfovibrionaceae, and Coriobacteriaceae, all of which were restored to normal levels by NACOS.

**Figure 7:**
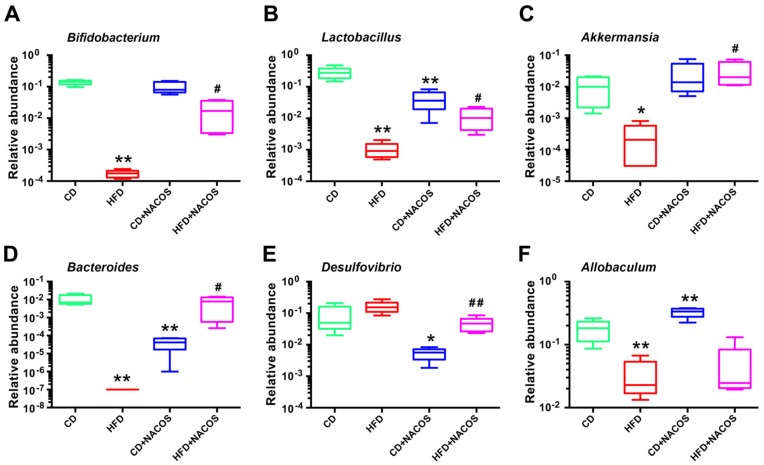
Effects of NACOS on the relative abundance of gut microbial community at genus level in HFD-fed mice, including *Bifidobacterium* (**A**), *Lactobacillus* (**B**), *Akkermansia* (**C**), *Bacteroides* (**D**), *Desulfovibrio* (**E**), and *Allobaculum* (**F**). Mice were fed with CD, HFD, CD + NACOS (1 mg/mL, in drinking water, about 200 mg/kg/day) or HFD + NACOS for five months. After the treatment, mice were euthanized and the fecal contents were collected for 16S rRNA sequencing. * *p* < 0.05, ** *p* < 0.01 compared to the CD group; ^#^ *p* < 0.05, ^##^ *p* < 0.01 compared to the HFD group.

Furthermore, several bacteria including *Bifidobacterium*, *Lactobacillus*, *Akkermansia*, *Bacteroides*, *Desulfovibrio*, and *Allobaculum* at the genus level were selected for further analysis. It was suggested that the abundances of four beneficial bacteria, *Bifidobacterium* (Figure 7A), *Lactobacillus* (Figure 7B), *Akkermansia* (Figure 7C), and *Bacteroides* (Figure 7D), were reduced in the HFD group (*p* < 0.05, vs. the CD group), which was significantly promoted after NACOS treatment (Figure 7A–D). NACOS also distinctly reduced the abundance of *Desulfovibrio* in HFD-fed mice (Figure 7E), but had no effect on *Allobaculum* (Figure 7F). Moreover, NACOS displayed similar restoring effects on the changes of other low-abundant genera in the HFD group (Figure S7).

### 2.7. Correlation between Gut Microbiota and Metabolic Parameters

**Figure 8:**
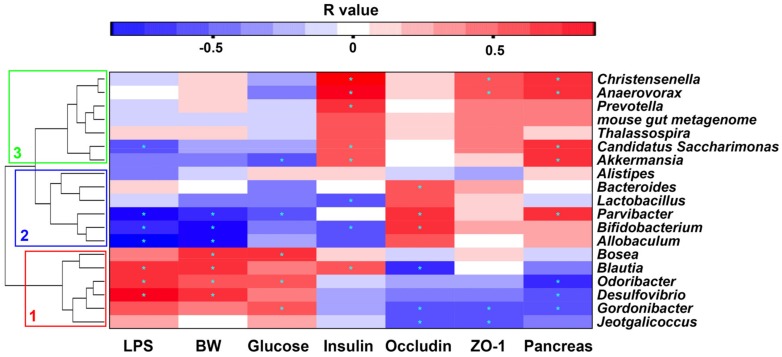
Correlation between metabolic biomarkers and relative abundance of intestinal bacteria at the genus level. The genera were distinctly divided into three clusters (cluster 1: red frame, cluster 2: blue frame, cluster 3: green frame). BW: body weight; LPS: lipopolysaccharide. The color of cells represents correlation *R* values from (–1)–0–1 (blue–white–red). Cells marked with an asterisk show significance following Spearman correlation for multiple comparisons, *p* < 0.05.

To further explore the correlation between specific bacterial taxa and metabolic parameters, we tabulated the Spearman correlation heat map and selected several tropical markers related to the tight junction (ZO-1 and occludin), inflammation (LPS), and glucose metabolism (Figure 8). There were three distinct clusters based on the strong correlation between genera and metabolic parameters. Cluster 1, including *Bosea*, *Blautia*, *Odoribacter*, *Desulfovibrio*, *Gordonibacter*, and *Jeotgalicoccus*, showed a positive relationship with LPS, glucose levels, and body weight, and negative a relationship with occludin, ZO-1, and pancreas weight. Cluster 2, including *Alistipes*, *Bacteroides*, *Lactobacillus*, *Parvibacter*, *Bifidobacterium*, and *Allcobaculum*, indicated the opposite effect as that of Cluster 1. Cluster 3, containing *Christensenella*, *Anaerovorax*, *Prevotella*, *mouse gut metagenome*, *Thalassospira*, *Candidatus Saccharimonas*, and *Akkermansia*, suggested a positive correlation with insulin levels and pancreas weight. 

**Figure 9:**
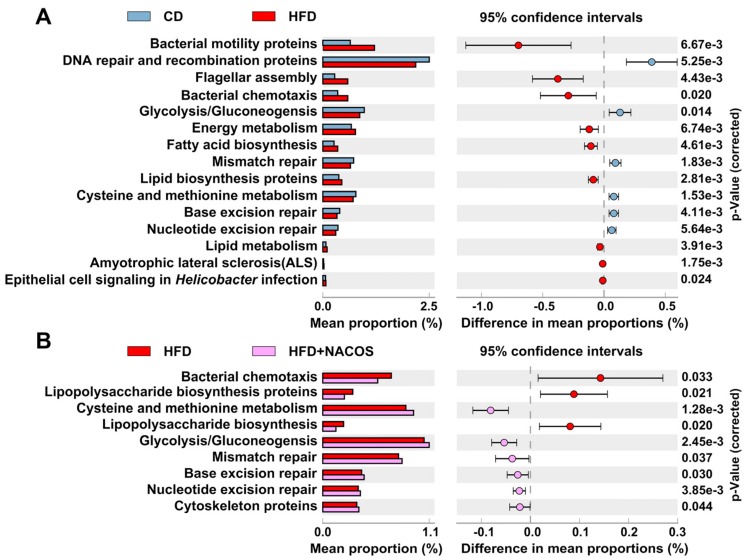
Prediction of changed KEGG pathways using PICRUSt analysis. (**A**) A total of 15 KEGG pathways were significantly changed in the HFD group compared with the CD group; (**B**) a total of nine KEGG pathways were significantly recovered in the HFD+NACOS group (bar plots on the left side displayed the mean proportion of each KEGG pathway. Dot plots on the right show the differences in mean proportions between the two indicated groups using *p*-values).

In order to gain a more fundamental understanding of the altered gut microbiota, we predicted their functional profiles using PICRUSt (Phylogenetic Investigation of Communities by Reconstruction of Unobserved States) analysis. As shown in Figure 9A, based on the composition of gut microbiota, 15 Kyoto Encyclopedia of Genes and Genomes (KEGG) pathways of gut microbiota were estimated to be affected in the HFD group. These pathways included bacterial motility (bacterial motility proteins, flagellar assembly, and bacterial chemotaxis), oxidative stress (DNA repair, mismatch repair) and energy metabolism (glycolysis/gluconeogensis, lipid metabolism, and fatty acid biosynthesis) (Figure 9A). Interestingly, it seems that NACOS treatment significantly influenced bacterial motility (bacterial chemotaxis), oxidative stress (nucleotide excision repair), energy metabolism (glycolysis/gluconeogensis), and inflammation process (lipopolysaccharide biosynthesis) (Figure 9B). 

## 3. Discussion

Thus far, NACOS has displayed a series of pharmacological effects, such as antimicrobial activity and protection against pathogen-induced infection [39]. As one of the major chitinous products, most recent studies focused on the biological activities of NACOS for its wide applications in various fields [40]. In this study, we, for the first time, demonstrated that NACOS attenuated HFD-induced metabolic syndrome, which may be related to the modulation of gut microbiota community.

Here, we proved that NACOS treatment, not only suppressed glucose intolerance, but directly lowered fasting blood glucose in HFD-fed mice (Figure 2A–C), indicating the improvement of glucose homeostasis in vivo. On the other hand, NACOS promoted the production of random plasma insulin in mice fed with a control diet or HFD. Though up-regulated insulin levels usually reflect impairment of insulin sensitivity, we speculate that the increase in insulin secretion should help to reduce the blood glucose of mice, instead of impairing insulin sensitivity, which can be indirectly proven by the inhibition of hyperglycemia and the improvement of glucose intolerance in HFD-fed mice after NACOS treatment. It was reported that COS stimulated glucose-inducible insulin expression of beta-cells in diabetic rats with streptozotocin injection [41]. Considering the promoting effect of COS on insulin production, it is possible that the anti-diabetic effects of NACOS may also be associated with increased insulin secretion and elevated proliferation of islets beta-cells in diabetic mice. This can be partly reflected by the increase of pancreatic mass in HFD-fed mice after NACOS treatment (Figure 1F), and this should be confirmed in future work.

In general, long-term intake of high-energy foods will increase the risk of metabolic syndrome, characterized by symptoms, such as hyperglycemia, dyslipidemia, obesity, and glucose intolerance. Since HFD feeding can simulate the energy-dense food of human beings (e.g., a Western diet and fast food) to a large extent, we fed mice with HFD for five months to induce metabolic disorders. We found that NACOS alleviated the dyslipidemia of HFD-fed mice by reducing triglyceride levels in blood and liver tissues (Figure S4) and blocking the transcription of several genes related to lipogenesis, adipocyte differentiation, and gluconeogenesis (Figure 3). In combination with reducing weight, NACOS displayed the potential to be a functional food or biological medicine to prevent the occurrence of metabolic diseases. Since inflammatory response has been demonstrated to be a key contributor to metabolic syndrome, we further examined the effects of NACOS on the expression of pro-inflammatory cytokines in adipose tissues. The results showed that NACOS dramatically reduced the mRNA levels of cytokines, including TNF-α, IL-6, and MCP-1, and also significantly decreased plasma concentrations of LPS in HFD-fed mice (Figure 4). The LPS in circulatory system originally came from Gram-negative bacteria via pathogenic invasion of the leaky gut barrier [42]. Indeed, in this study, we observed the reduction of major tight junction proteins (ZO-1 and occludin) and damage to the mucus layer of the proximal colon in HFD-fed mice, which were significantly suppressed by NACOS treatment (Figure 5). It was indicated that the strengthening of the intestinal barrier may be the reason for reduced LPS concentrations in blood and in the attenuated inflammation of adipose tissues of HFD-fed mice. 

Normally, the lifespan of mouse is two to three years, which means that mice aged six months can be still regarded as “young adults”. It was recently reported that the fecal microbial composition varied little in the first year of healthy rats (with 2.5–3 years of the lifespan) [43]. Therefore, in this study, the metabolic data of gut microbiota in mice were determined after five months of treatment (six months old).

Gut microbial composition can be changed by altered phyla ratios, which further enhances the pro-inflammatory potential of harmful intestinal bacteria [44]. There are contradictory conclusions regarding the change of Firmicutes/Bacteroidetes (F/B) ratios when the adiposity occurred. For example, Koropatkin et al. [45] reported that an increased ratio of F/B promoted the development of obesity in mice. In other studies, a low F/B ratio was identified in obese mice or people [46,47]. In our research, HFD feeding decreased the ratio of F/B, which was restored by NACOS treatment (Figure 6B). Further, at the family level, the reduced abundances of Porphyromonadaceae, Deferribacteraceae, Coriobacteriaceae, and the increased abundances of Rhodospirillaceae, Christensenellaceae, Bacteroidaceae, Lactobacillaceae, Bifidobacteriaceae, Verrucomicrobiaceae, and Erysipelotrichaceae, seen in HFD-fed mice, were also reversed by NACOS treatment (Figure 6C). The above results indicate that the direct modulating effects of NACOS on gut microbiota may play a critical role in the prevention of metabolic syndrome.

Evidence suggests that the increased abundances of *Bifidobacterium*, *Lactobacillus*, *Akkermansia muciniphila*, and *Bacteroides* were positively related to intestinal integrity, glucose tolerance, or attenuated obesity [2,9,14,48,49,50,51,52]. It is generally accepted that prebiotics usually promote the growth of beneficial intestinal bacteria, such as *Lactobacillus* and *Bacteroides* [53]. In this study, NACOS dramatically increased the abundances of *Bifidobacterium*, *Lactobacillus Akkermansia*, and *Bacteroides* in HFD-fed mice (Figure 7). In addition, the Gram-negative *Desulfovibrio* genus is commonly increased following a fat-enriched diet [54], and is responsible for inflammation due to its lipid A structures of LPS [55]. In accordance with these reports, our study showed that NACOS significantly reduced the abundance of the *Desulfovibrio* genus in both the CD group and the HFD group (Figure 7E). Noticeably, NACOS evidently reduced the abundance of *Bacteroides* and increased that of *Allobaculum* belonging to Firmicutes phylum in the CD group (Figure 7D,F). Similar results were also observed in studies on ginseng polysaccharides and inulin-type fructans [56,57], which are widely used prebiotics. This further demonstrated the complexity of gut microbiota in modulating the health of a host.

The correlation heat map between intestinal taxa and metabolic parameters indicated the different effects of bacteria. Our results suggested that *Desulfovibrio* had a close relationship with LPS biosynthesis pathway (Figure 8), so the reduced abundance of *Desulfovibrio* by NACOS treatment may contribute to reduction of LPS biosynthesis (Figure 9B). 

The prediction of functional profiles, based on gut microbiota composition, was consistent with our experimental result that less LPS was detected in the plasma of HFD-fed mice with NACOS treatment. The PICRUSt analysis predicted that altered gut microbiota by NACOS showed less bacterial motility, LPS production, and oxidative stress. Our experimental results indicate that the ultimate outcome of NACOS treatment was the improvement of metabolic syndrome in HFD-fed mice (Figure 9).

In some of the studied parameters, NACOS had a slight opposite effect between CD + NACOS group and HFD + NACOS such as abdominal fat tissue (Figure 1E), GTT AUC (Figure 2C) and plasma insulin (Figure 2D). Usually, some polysaccharides or oligosaccharides displayed two-way regulatory effects on the host, for example, the inflammation-stimulating effects under physiological conditions and the anti-inflammatory effects under pathological conditions. This may partly explain why NACOS displayed a different effect on ND-fed mice as compared to that on HFD-fed mice. In addition, the Spearman correlation analysis in Figure 8 suggested that the parameters including LPS, BW, glucose and insulin were strongly correlated with the intestinal microbiota. And the levels of the above parameters were in accordance with the changes of *Lactobacillus* (Figure 7B), *Bacteroides* (Figure 7D), *Desulfovibrio* (Figure 7E), and *Allobaculum* (Figure 7F) between four experimental groups. 

## 4. Materials and Methods 

### 4.1. Reagents

Chitin oligosaccharide with the polymerization degree 2–6 was prepared in our laboratory (Figure S3). Antibodies against ZO-1, occludin, and β actin were purchased from Santa Cruz Biotechnology (Santa Cruz, CA, USA). Other antibodies, including horseradish peroxidase (HRP)-conjugated goat anti-rabbit IgG and HRP-conjugated goat anti-mouse IgG, were purchased from Cell Signaling Technology (Beverly, MA, USA). All other regular chemicals used in this study were of the highest chemical grade. 

### 4.2. Mouse Treatment

Male C57BL/6J (20 ± 2 g, six weeks old) mice were purchased from Model Animal Research Center of Nanjing University (Nanjing, China). Mice were given water and food ad libitum throughout the experiment. After acclimation for one week at 22 ± 1 °C with a 12:12-h dark–light cycle, 20 mice were randomly divided into four groups (*n* = 5): normal chow diet (CD) group, high-fat diet (HFD) group, CD + NACOS (1 mg/mL in drinking water, about 200 mg/kg/d) group and HFD + NACOS group. The standard CD contained 20% protein, 70% carbohydrate, and 10% fat, whereas the HFD contains 20% protein, 35% carbohydrate, and 45% fat. Both diets used in the experiment were purchased from Aoke Xieli Co., Ltd. (Beijing, China). Detailed ingredients of the diets can be found in Table S1. During the treatment period, body weight, and fasting glucose were measured frequently. Drinking water and diet consumption were recorded every other week. After treatment for five months, all mice were euthanized. Mouse sera were isolated and major tissues were collected, including liver, abdominal adipose tissues, pancreas, intestinal tract, and fecal samples. All samples were stored at −80 °C for further experiments. 

The procedure for animal experiments was approved by the Animal Ethical Experimentation Committee of Institute of Process Engineering, Chinese Academy of Sciences (permission number: SYXK2015-0002) and in accordance with the National Act on Use of Experimental Animals (China).

### 4.3. Glucose Tolerance Test and ELISA Assay

A glucose tolerance test (GTT) was carried out at the end of the experiment. Briefly, after the mice fasted overnight, blood glucose was measured at 0 min, 15 min, 30 min, 60 min, and 120 min after intraperitoneal injection of glucose (2 g/kg body weight). Blood was collected from the tail vein, and the glucose concentration of each sample was determined using a blood glucometer (Roche Diagnostics, Basel, Switzerland). 

Plasma insulin levels were detected using a mouse ultrasensitive insulin ELISA kit (ALPCO, USA), and the plasma endotoxin concentration was measured using a mouse LPS ELISA kit (BlueGene Biotech, China) following the manufacturer’s protocol. The concentrations of triglyceride and high-density lipoprotein cholesterol in the serum and liver tissues were determined using a Cobas 8000 modular series analyzer (Roche Diagnostics, USA). 

### 4.4. RNA Extraction and Quantitative Real-Time PCR (qRT-PCR)

Total RNA was extracted from abdominal adipose tissue using TRIzon Reagent (CWBio, Beijing, China), and 5 µg of RNA was transcribed to cDNA following the protocol of the HiFiScript cDNA synthesis kit (CWBio, Beijing, China). Polymerase reaction was performed using a SYBR Select Master Mix (Applied Biosystems,Foster City, CA, USA) with the following thermal cycle conditions: 95 °C for 5 min; 40 cycles of amplification (95 °C for 15 s, 60 °C for 60 s). The fluorescence intensity was captured and analyzed using a 7500 Fast Real-Time PCR system (Applied Biosystems, Foster City, CA, USA). The mRNA expression levels of stearoyl-CoA desaturase-1 (Scd-1), CCAAT/enhancer-binding protein alpha (C/EBPα), peroxisome proliferator-activated receptor gamma (PPARγ), peroxisome proliferator-activated receptor alpha (PPARα), glucose-6-phosphatase (G6Pase), phosphoenolpyruvate carboxykinase (PEPCK), leptin, tumor necrosis factor alpha (TNF-α), interleukin 6 (IL-6), and monocyte chemoattractant protein 1 (MCP-1) were normalized using β-actin as an internal control, and the primer sequences are given in Table S2. The relative fold difference of mRNA was calculated using the comparative Ct method and is expressed as 2^−ΔΔCt^.

### 4.5. Western Blotting 

One hundred micrograms of intestinal tissue were homogenized with RIPA buffer (Cell Signaling, MA, USA). About 40 μg of protein lysates was fractionated on 8% sodium dodecyl sulfate-polyacrylamide gel (SDS-PAGE) and transferred to polyvinylidene fluoride membrane (Millipore, Bedford, MA, USA) for Western blot analysis. Membranes were probed with primary antibodies against ZO-1, occludin and β-actin overnight at 4 °C, followed by incubation with horseradish peroxidase-conjugated secondary antibody. Protein bands were captured using enhanced chemiluminescence (ECL) (Cell Signaling Technology, Danvers, MA, USA) and densitometry analysis was conducted using Image J2x software (National Institute of Health, Bethesda, MD, USA).

### 4.6. Immunofluorescent Staining 

For immunofluorescent staining, the proximal colon segments, fixed in 4% paraformaldehyde, were sliced and trimmed into serial sections. After being deparaffinized in xylene and rehydrated through ascending ethanol series, the slides were permeabilized with 0.1% Triton-X 100 for 5 min, blocked with 10% goat serum in PBST (PBS with 0.05% Tween 20) for 1 h, and incubated with WGA-FITC (1:1000) at 4 °C overnight, which can specifically identify the glycans of mucin. Washed with PBST, the coverslips were mounted with anti-fade reagent with 4′, 6′-diamidino-2-phenylindole (DAPI) (Life Technologies, Waltham, MA, USA). Images were acquired using a Leica DFC310 FX digital camera connected to a Leica DMI4000 B light microscope (Wetzlar, Germany). The contents of mucin glycoproteins were reflected by the fluorescence intensity of each slide.

### 4.7. 16S rRNA Microbial Community Analysis

Mouse fecal DNA was extracted using a FastDNA™ SPIN Kit (MP Biomedicals, Santa Ana, CA, USA). The 16S rRNA gene comprising V3–V4 regions was amplified using a forward primer 338F (5′-ACTCCTACGGGAGGCAGCAG-3′) and a reverse primer 806R (5′-GGACTACHVGGGTWTCTAAT-3′). PCR reactions were performed in triplicate: 50 μL mixture containing 2 μL of each primer (10 μM), 4 μL of dNTPs (2.5 mM), 5 μL of 10 × Pyrobest Buffer, 0.3 μL of Pyrobest DNA Polymerase (2.5 U/μL) and 30 ng of DNA sample. The amplification protocol was as follows: 95 °C for 5 min, 25 cycles at 95 °C for 30 s, 56 °C for 30 s, 72 °C for 40 s and a final extension at 72 °C for 10 min. Amplicons were extracted from 2% agarose gels and purified using the AxyPrep DNA Gel Extraction Kit (Axygen Biosciences, Union City, CA, USA). Purified amplicons were paired-end sequenced on the MiSeq Illumina MiSeq platform by Allwegene Technology Inc. (Beijing, China), and 16S rRNA gene sequences were analyzed using the Quantitative Insights Into Microbial Ecology (QIIME) software (Version 1.8, http://qiime.org/) package with the following criteria: 1) trimming sequences over a 50 bp sliding window and discarding truncated reads shorter than 200 bp; 2) less than two nucleotide mismatches in primer matching; 3) assembling the paired reads according to their overlap sequence with a mismatch rate < 0.1; 4) chimera sequences were identified and removed using Usearch software (version 8.1.1861, http://www.drive5.com/usearch/). All sequences were used for the comparison of relative abundance of bacterial taxa, and were clustered into operational taxonomic units (OTUs) according to a 97% similarity. The taxonomy of each 16S rRNA gene sequence was analyzed using UCLUST (version 1.2.22, http://www.drive5.com/uclust/downloads1_2_22q.html) against the Silva119 16S rRNA database using a confidence threshold of 90%. PICRUSt analysis was conducted as performed previously [58].

### 4.8. Statistical Analysis

Data are presented as means ± SD. Differences between two groups were analyzed using an unpaired Student’s *t*-test. Datasets that involved more than two groups were assessed with one-way analysis variance (ANOVA), along with the Tukey–Kramer test. *p* < 0.05 was regarded as statistically significant. Regular analysis was carried out using GraphPad Prism (version 7.0a, GraphPad Software Inc., San Diego, CA, USA). 

## 5. Conclusions

In this study, NACOS feeding maintained the stability of intestinal homeostasis in HFD-treated mice, which was accompanied by an elevated abundance of beneficial bacteria (*Bifidobacterium*, *Lactobacillus*, *Akkermansia*, *Bacteroides*, etc.), the reduced population of pro-inflammatory bacteria (*Desulfovibrio*) and enhanced gut integrity. Further, NACOS significantly decreased the LPS concentration in the circulation system, alleviated the occurrence of dyslipidemia and inflammation in adipose tissues, and, thus improved hyperglycemic symptoms in mice.

## Supplementary Materials

The following are available online at http://www.mdpi.com/1660-3397/16/2/66/s1. Table S1: Ingredient of control diet (CD) and high-fat diet (HFD) in the mouse experiment; Table S2: Primers used in this study; Table S3: Recovery rates of spiking control for LPS ELISA assay; Figure S1: Standard curve of LPS ELISA assay, and LPS with different concentrations was used as spiking control; Figure S2: Intra- & inter individual assay variation (CV) of LPS ELISA kit (E03L0268, BlueGene Biotech, China); Figure S3: LC-MS spectrum of NACOS; Figure S4: Improvement of NACOS on the lipid levels in liver and blood of HFD-fed mice; Figure S5: Inhibition of NACOS on lipid accumulation in liver tissues of HFD-treated mice; Figure S6: Suppressive effect of NACOS on increment of adipocyte size in abdominal adipose tissues of HFD-treated mice; Figure S7: Effect of NACOS on the relative population of gut microbial bacteria with low abundance in mice after HFD treatment at genus level.
