# Supplementary material for: Chitin Oligosaccharide Modulates Gut Microbiota and Attenuates High-Fat-Diet-Induced Metabolic Syndrome in Mice
Source: Mar Drugs. 2018 Feb 19;16(2):66. doi: 10.3390/md16020066 (PMC5852494; doi:10.3390/md16020066)
Supplement: Supplementary file 1 [file marinedrugs-16-00066-s001.pdf]

Chitin Oligosaccharide Modulates Gut Microbiota and Attenuates High-Fat-Diet-Induced Metabolic Syndrome in Mice

Supplementary Information

Files in this data supplement

Supplementary Materials and Methods

Supplementary tables

Supplementary figures

Supplementary Materials and Methods

Preparation of Chitin Oligosaccharides (NACOS)

Chitin was extracted from crab shells, and the chitosan sample was made by Qingdao Kading Co., China. NACOS was made by our laboratory. Briefly, 5 g of chitosan oligosaccharides (300–1700 Da) were dissolved in 50 mL of water and reacted with a mixture of 4.37 ml acetic anhydride and 0.1 g of 4-dimethyl aminopyridine in 3 mL of methanol at 60 °C for 4 h. Next, the reaction mixture was precipitated with five-fold acetone (v/v). After further washing with acetone, three times, the gray–white powder product, i.e., NACOS, was dried under a vacuum for 2 h. Finally, the acetylation degree (97%) and polymeration degree (2–6) of NACOS was identified by LC-MS analysis (Figure S1).

Oil Red O staining

Liver tissues fixed in 4% paraformaldehyde were sliced and trimmed into serial sections. After being deparaffinized in xylene and rehydrated through ascending ethanol series, sections were stained with Oil Red O solution for 15 min and then counterstained with hematoxylin for 1 min. The slides were observed under a Leica DMI4000 B light microscope (Wetzlar, Germany).

Supplementary tables

Table S1. Ingredients of control diet (CD) and high-fat diet (HFD) in the mouse experiment.

Ingredient	Control Diet		High-Fat Diet	
	Gram	Kcal	Gram	Kcal
Casein, 80 Mesh	200	800	200	800
L-Cystine	3	12	3	12
Corn Starch	315	1260	72.8	291
Maltodextrin 10	35	140	100	400
Sucrose	350	1400	172.8	691
Cellulose, BW200	50	0	50	0
Soybean Oil	25	225	25	225
Lard	20	180	177.5	1598
Mineral Mix S10026	10	0	10	0
Dicalcium Phosphate	13	0	13	0
Calcium Carbonate	5.5	0	5.5	0
Potassium Citrate, 1 H_2O	16.5	0	16.5	0
Vitamin Mix V10001	10	40	10	40
Choline Bitartrate	2	0	2	0
FD&C Red Dye #40	0.05	0	0.05	0

Table S2. Primers used in this study.

Primers	Forward Primer	Reverse Primer
Actin	AGGTGACAGCATTGCTTCTG	GCTGCCTCAACACCTCAAC
IL-6	GAAACCGCTATGAAGTTCCTCTCTG	TGTTGGGAGTGGTATCCTCTGTGA
TNF-α	AGGGTCTGGGCCATAGAACT	CCACCACGCTCTTCTGTCTAC
MCP-1	GGGATCATCTTGCTGGTGAA	AGGTCCCTGTCATGCTTCTG
Scd-1	TTCTTGCGATACACTCTGGTGC	CGGGATTGAATGTTCTTGTCGT
C/EBPα	CAAGAACAGCAACGAGTACCG	GTCACTGGTCAACTCCAGCAC
PPARα	CCCTGCCATTGTTAAGACC	TGCTGCTGTTCCTGTTTTC
PPARγ	CTCCAAGAATACCAAAGTGCGA	GCCTGATGCTTTATCCCCACA
G6Pase	CGACTCGCTATCTCCAAGTGA	GTTGAACCAGTCTCCGACCA
PEPCK	CTGCATAACGGTCTGGACTTC	CAGCAACTGCCCGTACTCC
Leptin	GCCAGGCTGCCAGAATTG	CTGCCCCCCAGTTTGATG

Table S3. Recovery rates of spiking control for LPS ELISA assay.

LPS(ng/ml)	OD Value	Concentration (ng/ml)	Recovery Rate (%)
0.0	1.2242	–0.3	
10.0	1.1803	9.8	98.2
25.0	1.1318	24.3	97.2
50.0	1.0340	52.8	105.7
100.0	0.8573	104.8	104.8
250.0	0.3623	250.5	100.2

Supplementary figures

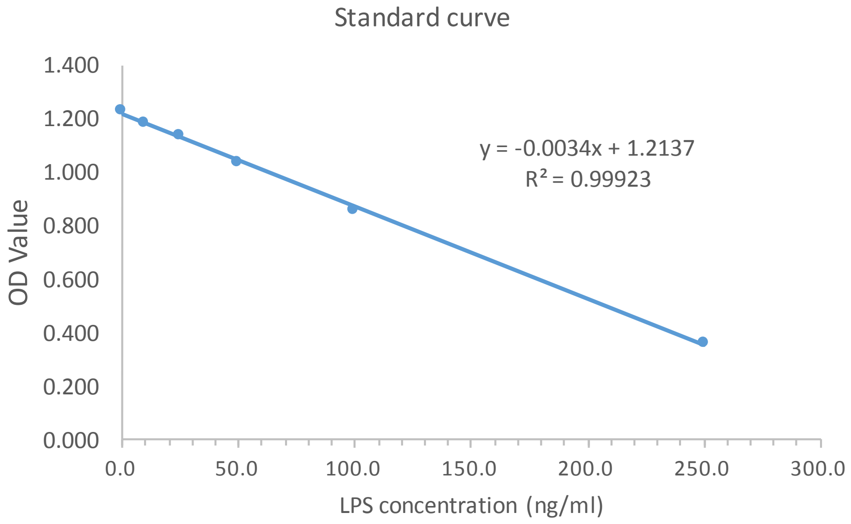

Figure S1. Standard curve of LPS ELISA assay, and LPS with different concentrations used as spiking control.

10. CERTIFICATE OF ANALYSIS

1) In the same lot CV%: 4.4, 5.6
2) Different lot CV%: 6.6, 7.9
3) Spike Recovery: 94-103%
4) Linearity:

	Range %
1:2	96 – 101
1:4	93 - 107
1:8	92 - 100
1:16	96 - 108

5) Sensitivity: The sensitivity in this assay is 1.0 ng/ml.

Figure S2. Intra- & inter individual assay variation (CV) of LPS ELISA kit (E03L0268, BlueGene Biotech, China).

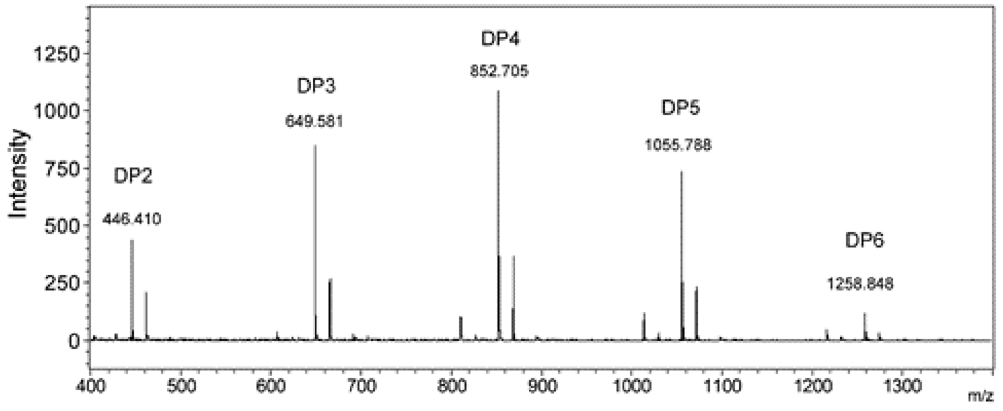

Figure S3. LC-MS spectrum of NACOS. DP, degree of polymerization**.**

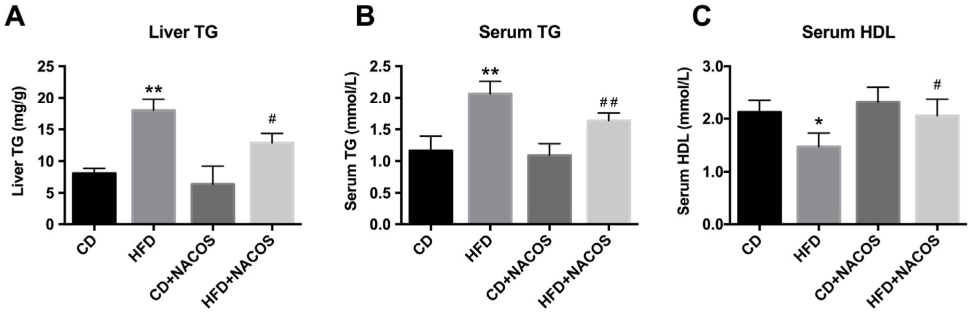

Figure S4. Improvement of NACOS on lipid level in liver and blood of HFD-fed mice. Mice were fed with CD, HFD, CD + NACOS (1 mg/ml, in drinking water), or HFD + NACOS) for five months. After that, mice were sacrificed, and blood and liver tissues were collected. Liver triglyceride (**A**), serum triglyceride (**B**), and serum high-density lipoprotein (**C**) were determined. TG, triglyceride; HDL, high-density lipoprotein-cholesterol. Data are represented as means ± SD (n = 5). *P < 0.05, **P < 0.01 compared to CD group; $^{\#}P$ < 0.05, $^{\#\#}P$ < 0.01 compared to HFD group.

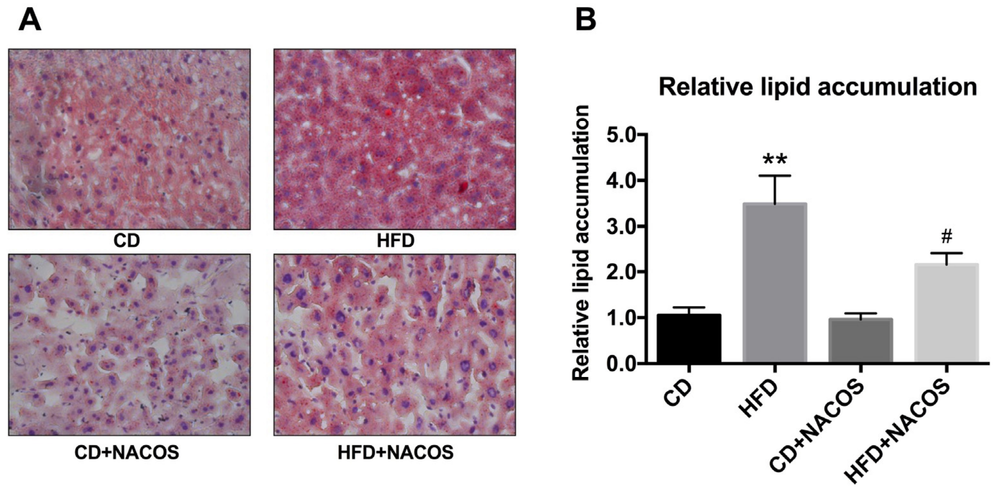

Figure S5. Inhibition of NACOS on lipid accumulation in liver tissues of HFD-treated mice. (**A**) Histological photograph of Oil Red O staining; (**B**) quantification of lipid accumulation in liver tissues. Red, lipid droplets; blue, nuclei. Data are represented as means ± SD (n = 5). *$P < 0.05$, **$P < 0.01$ compared to CD group; $^{\#}P < 0.05$ compared to HFD group.

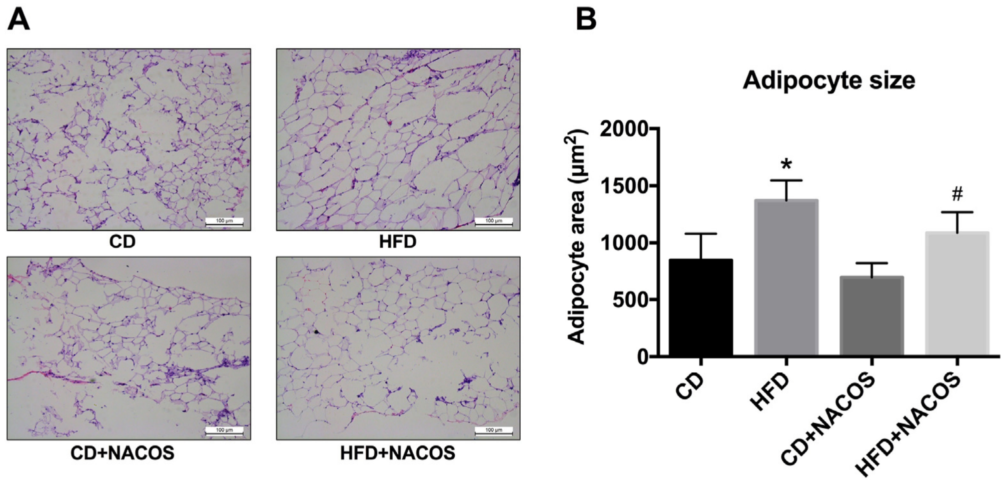

Figure S6. Suppressive effect of NACOS on increment of adipocyte size in abdominal adipose tissues of HFD-treated mice. (**A**) Histological photograph of H&E staining; (**B**) corresponding quantitative results of (**A**). Data are represented as means ± SD (n = 5). *$P < 0.05$ compared to CD group; $^{\#}P < 0.05$ compared to HFD group.

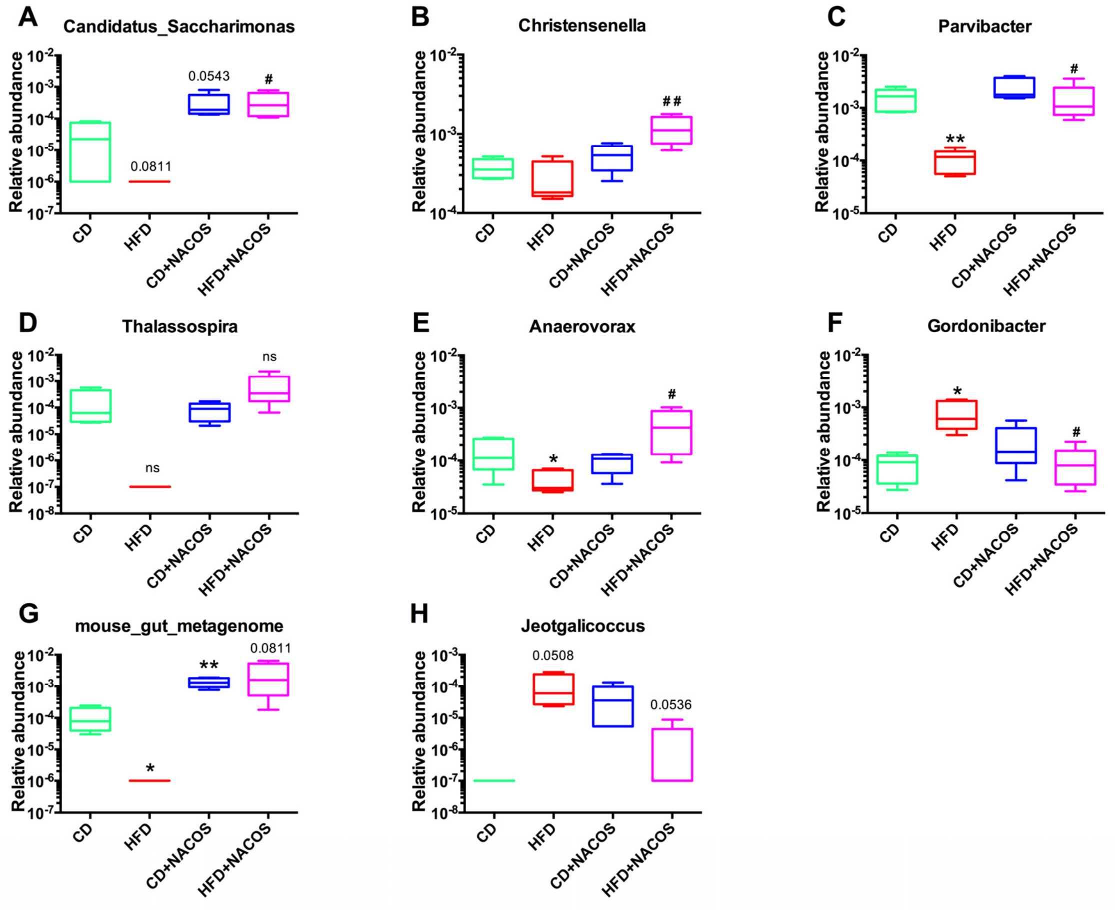

Figure S7. Effect of NACOS on the relative population of gut microbial bacteria with low abundance in mice after HFD treatment at genus level, including *Candldatus_Saccharimonas* (**A**), *Christensenella* (**B**), *Parvibacter* (**C**), *Thalassospira* (**D**), *Anaerovorax* (**E**), *Gordonibacter* (**F**), *Mouse_gut_metagenome* (**G**), and *Jeotgalicoccus* (**H**). Data are represented as means ± SD (n = 5). *P < 0.05, **P < 0.01 compared to CD group; #P < 0.05, ##P < 0.01 compared to HFD group.
